# Influence of Haemolysis on the Mineral Profile of Cattle Serum

**DOI:** 10.3390/ani11123336

**Published:** 2021-11-23

**Authors:** Belén Larrán, Marta Miranda, Carlos Herrero-Latorre, Lucas Rigueira, Víctor Pereira, María Luisa Suárez, Marta López-Alonso

**Affiliations:** 1 Department of Anatomy, Animal Production and Clinical Veterinary Sciences, Faculty of Veterinary, Campus Terra, University of Santiago de Compostela, 27002 Lugo, Spain; belen.larran.franco@usc.es (B.L.); lucas.rigueira@usc.es (L.R.); maruska.suarez@usc.es (M.L.S.); 2 Rof-Codina Veterinary Teaching Hospital, Faculty of Veterinary, Campus Terra, University of Santiago de Compostela, 27002 Lugo, Spain; 3 Research Institute on Chemical and Biological Analysis, Analytical Chemistry, Nutrition and Bromatology Department, Faculty of Sciences, Campus Terra, University of Santiago de Compostela, 27002 Lugo, Spain; carlos.herrero@usc.es; 4 Department of Animal Pathology, Faculty of Veterinary, Campus Terra, University of Santiago de Compostela, 27002 Lugo, Spain; victor.pereira@usc.es (V.P.); marta.lopez.alonso@usc.es (M.L.-A.)

**Keywords:** haemolysis, mineral elements, serum, cattle, ICP-MS

## Abstract

**Simple Summary:**

The results of blood tests routinely used in clinical chemistry can be altered by haemolysis, the disruption of red blood cells. Haemolysis of serum samples is recognized to be the leading cause of preanalytical errors in clinical laboratories. The influence of haemolysis must be specifically studied for each analyte and species of clinical interest, as it is often not known how serum samples are affected. Little is known about the potential alterations in the concentrations of mineral elements in haemolyzed serum in general and the phenomenon has not been specifically studied in bovine serum samples. We investigate how haemolysis affects the mineral content of bovine samples.

**Abstract:**

Haemolysis of serum samples is the leading cause of preanalytical errors in clinical laboratories. Little is known about the potential alterations in the concentrations of mineral elements in haemolyzed serum and the phenomenon has not been specifically studied in bovine serum samples. We investigate how haemolysis affects the mineral content of bovine samples. We used ICP-MS to measure the concentrations of 12 mineral elements (Ca, Co, Cr, Cu, Fe, Mg, Mn, Mo, Ni, P, Se and Zn) in bovine whole blood, serum and gradually haemolyzed samples and observed significant differences between the different types of samples, particularly in the Fe and Zn concentrations. However, in practice, the high interindividual variability makes it difficult to establish whether a given value corresponds to normal or haemolyzed samples. In response to this problem, we propose to consider that a result is significantly biased when the haemolysis threshold (the degree of haemolysis above which the concentration of an element in serum is significantly altered) of a given element is surpassed. The haemolysis threshold values for the different elements considered were found as follows: 0.015 g Hb L^−1^ for Fe, 2 g for Zn, 4 g for Cr and 8 g for Ca, Se and Mo.

## 1. Introduction

Minerals are inorganic elements that participate in almost all biochemical processes in living organisms and play a critical role in animal health and production [1]. Minerals have structural, physiological, catalytic and regulatory functions and are involved in tissue growth, cell replication and differentiation, energy and oxidative metabolism and immunity, among other vital processes [1,2]. Twenty-five mineral elements are considered essential to animals [3] and must be obtained through the diet.

Mineral deficiencies, imbalances and toxicities can lead to obvious clinical disorders or can be manifested as subclinical processes that affect the profitability of livestock through decreased growth, reproduction and production rates [4,5]. Therefore, optimal mineral intake is important to maintain cattle health and also to maximize production. As most essential mineral elements have large safety margins, supplementation in feed has become routine practice. Therefore, mineral deficiencies and imbalances are less frequent nowadays and are almost exclusively reported in pasture-based farming in which concentrate feed is limited or avoided, as in the emergent sustainable production systems [6]. However, excessive mineral supply causes environmental pollution and has become of concern in the European Union, especially in relation to copper (Cu) and zinc (Zn) [7,8]; thus, mineral supplements must be carefully adjusted to meet the physiological needs of livestock. These requirements should be tailored according to the species, breed, sex, age, production phase, food properties and total intake [1,9,10]. Moreover, monitoring the mineral profile can provide useful information in relation to human nutrition and health, as animal products are one of the main sources of essential mineral intake and toxic element residues in the human diet [11].

Blood is the most commonly used and convenient sample for preliminary determination of the mineral profile of herds, as collection is simple and non-lethal and modern atomic spectrometry techniques provide precise and inexpensive mineral determinations at low concentrations [12,13]. Whole blood is seldom used and plasma and serum are considered suitable and interchangeable for most elements [14,15]. Almost all available data regarding physiological mineral concentrations in cattle refer to serum [16,17].

Haemolysis is the leading cause of sample rejection in laboratories and, although little is known about its influence in mineral determination, it is widely recognized to bias the results of most routine analyses [18,19,20]. Blood cells may be disrupted for pathological reasons or due to sample handling, with the latter being the most frequent cause of preanalytical errors [21]. The intracellular and serum concentrations of some mineral elements are known to differ; thus, mineral profiles can potentially be altered by haemolysis. However, as broad comparative studies of mineral concentrations in cattle whole blood and serum/plasma are scarce, the influence of haemolysis cannot be accurately predicted. While the release of blood cell contents could increase the concentrations of some minerals, as is known to occur with iron (Fe), Zn, selenium (Se), magnesium (Mg) and manganese (Mn) [13,17,22,23,24], the concentrations of other elements, such as Cu [25], may decrease due to dilution of the sample if the intracellular concentration is lower. Nonetheless, if the variation in mineral concentration is not significant, the haemolyzed sample may be suitable for analysis and unnecessary sample rejection could be avoided.

In the present study, the mineral contents of whole blood, serum and in vitro-graded haemolyzed serum samples were determined by inductively coupled plasma-mass spectrometry (ICP-MS). The first objective of the study is to determine any significant differences between mineral concentrations measured in whole blood and in serum. The second objective is to establish a haemolysis acceptability threshold (HTr) to enable laboratory technicians and clinicians to decide whether a serum sample would yield reliable results for measuring mineral contents.

## 2. Materials and Methods

### 2.1. Sample Collection and Preparation

Blood samples were obtained by jugular venepuncture in ten healthy Holstein-Friesian cows used for teaching clinical examination methods and housed in the Rof-Codina Veterinary Teaching Hospital, Faculty of Veterinary Medicine, University of Santiago de Compostela (Spain). Data collection was carried out according to Directive 2010/63/EU on the protection of animals used for scientific purposes [26] and the trial complied with the Spanish legislation on animal care [27]. The procedures were supervised by the Bioethics Committee of the Rof-Codina Veterinary Teaching Hospital, University of Santiago de Compostela (Spain).

Three types of blood samples were collected from each cow, to provide serum and whole blood for mineral analysis and to prepare the haemolysate for haematological analysis. Whole blood samples were collected in 9 mL serum tubes (Vacuette^®^, CAT Serum Clot Activator; Greiner Bio-One, Kremsmünster, Austria) and centrifuged within 4 h of collection, at 1500× *g* for 15 min, to yield serum for mineral analysis and graded haemolysed sample preparation. The tubes of serum were stored at −20 °C for further analyses. Whole blood samples collected in 9 mL tubes containing sodium heparin (Vacuette^®^, NH sodium heparin, Greiner Bio-One, Kremsmünster, Austria) were used for the mineral analysis and to produce the haemolysate by freezing (−20 °C). The haematological analysis was performed in whole blood samples collected in 6 mL tubes containing ethylenediaminetetraacetic acid (EDTA) (Vacuette^®^, K2E EDTA K2, Greiner Bio-One, Kremsmünster, Austria).

To prepare the graded haemolysis samples, each serum sample was divided into seven subsamples that were spiked with increasing amounts of haemolysate (0.0%, 0.2%, 0.5%, 1.0%, 2.5%, 5.0% and 10%) (Table 1). The haemolysis degree (HD), expressed as grams of haemoglobin (Hb) per litre, in each serum haemolysate-spiked sample was calculated from the concentration of Hb of each whole blood sample determined in an automated blood cell counter.

### 2.2. Apparatus and Reagents

Complete blood counts were conducted using an automated blood cell counter (ProCyte Dx, IDEXX Laboratories, Westbrook, ME, USA), based on a combination of fluorescence laser flow cytometry and laminar flow impedance technologies. The haematological parameters were normal in all of the cows [28]. The mean Hb concentration was 11.2 g dL^−1^, in a range of 10.2–11.9 g dL^−1^.

All solutions in this work were prepared with ultrapure Milli-Q water (Millipore Corp., Bedford, MA, USA). The stock standard solutions (1000 mg L^−1^) of the different elements analysed were prepared using ultrapure grade reagents (CertiPUR^®^, Merck, Poole, UK). Hyperpur^®^ nitric acid (69% *w*/*v*), used for sample digestion, was purchased from Panreac (Barcelona, Spain). Hydrogen peroxide (33% *w*/*v*) was also provided by Panreac. For ICP-MS quality control, the certified reference material (CRM) NIST SRM-1598a of inorganic constituents in animal serum was acquired from the National Institute for Standards and Technology (NIST) (Gaithersburg, MD, USA). The polypropylene tubes used for preparation of samples and standards were soaked in 10% Hyperpur HNO_3_ for at least 24 h, rinsed with ultrapure water and dried before use. The sample tubes were tested and found to be free of trace elements.

ICP determinations were carried out using an Agilent 7700x ICP-MS system (Agilent Technologies, Tokyo, Japan) equipped with collision/reaction cell interference reduction technology. The continuous sample introduction system consisted of an autosampler, a Scott double-pass spray chamber (Agilent Technologies, Tokyo, Japan), a glass concentric MicroMist nebuliser (Glass Expansion, West Melbourne, Australia), a quartz torch and nickel cones (Agilent Technologies, Tokyo, Japan). The element concentrations were quantified using a MassHunter Work-Station Software for ICP-MS (version A.8.01.01 Agilent Technologies, Inc. 2012, Tokyo, Japan). Sample digestions were performed in a microwave-assisted digestion system (Ethos Plus, Milestone, Sorisole, Italy).

### 2.3. Analytical Procedures

Whole blood and serum samples were subjected to acid digestion prior to ICP-MS analysis. In the case of whole blood, microwave-assisted digestion was applied. Briefly, 1 mL of sample (whole blood) was transferred to a polypropylene tube, to which 3 mL of concentrated nitric acid (69%) and 1 mL of ultrapure Milli-Q (Millipore Corp., Bedford, MA, USA) were then added. The mixtures were homogenized and processed in the microwave digestion system (Ethos Plus, Milestone, Sorisole, Italy). Digested samples were transferred to 25 mL flasks and ultrapure water was added to complete the volume. In the case of serum samples, a single digestion procedure was used, as previously described [12]. Briefly, 1 mL of each serum sample was transferred to polypropylene tubes, to which 1 mL of Hyperpur nitric acid (69%) and 0.5 mL of hydrogen peroxide (33%) were then added. The tubes were then placed in a thermostatic block at 60 °C for at least 2 h. The tubes were cooled and the digested samples were diluted to 5 mL with ultrapure Milli-Q water and centrifuged for 5 min at 2000 rpm. The supernatant was transferred to 5 mL polypropylene tubes.

Multi-element determination was performed by ICP-MS for twelve elements: calcium (Ca), cobalt (Co), chromium (Cr), copper (Cu), iron (Fe), magnesium (Mg), manganese (Mn), molybdenum (Mo), nickel (Ni), phosphorus (P), selenium (Se) and zinc (Zn). Immediately before the analysis of whole blood or serum samples, calibration curves (in the range from 0.2 to 10,000 μg L^−1^) were prepared daily from fresh standard solutions. In all cases, the linear responses obtained from the ICP-MS instrument were straight lines with zero intercept, with correlation coefficients were higher than 0.999 and the relative standard deviations (RSD) were lower than 5%. An analytical quality control program was also applied throughout the study. The main results are summarized in Table 2. Analytical blanks were examined for all batches and the limit of detection (LOD) was calculated as 3 times the standard deviation of the blanks. The concentrations were above the LOD in all samples. The accuracy of the determinations was verified by measuring a certified reference material provided by NIST (Animal serum 1598a) and spiked whole blood and serum samples at the appropriate concentration levels. Overall, good recovery of both the CRM and the spiked samples was achieved (Table 2).

### 2.4. Estimation of the Haemolysis Threshold Affecting Mineral Concentrations in Serum

For a specified mineral element, the haemolysis threshold (HTr) is defined as the haemolysis degree (expressed in g Hb L^−1^) from which there is a significant change (increase or decrease) in the concentration of that mineral in the serum. In order to estimate this value, the mean concentrations of minerals in haemolysate and serum, the mean concentrations of Hb in the whole blood samples of the individuals and the analytical variation of the ICP method used for the serum analysis (calculated as the coefficient of variation in Luna et al. [12]) were considered. The HTr was calculated as the haemolysis degree necessary for the concentration of an element to be modified (above or below) by a greater amount than the analytical variation of the technique used for that element. Thus, in this case, the variation can be detected by the analytical technique and calculated using the following equation:

$$\frac{V_{S}{\left[E\right]}_{S}+V_{H}{\left[E\right]}_{H}}{V_{F}}={\left[E\right]}_{S}+{\left[E\right]}_{S}\;\frac{CV}{100}$$

to give

$$V_{H}=\frac{{\left[E\right]}_{S}\;\frac{CV}{100}}{{\left([E\right]}_{H}-{\left[E\right]}_{S})}{\;V}_{F}$$

and then

HTr (g Hb L^−1^) = *V_H_* [Hb]

where $V_{S}$ and ${\left[E\right]}_{S}$ are the volume of serum and the concentration of the mineral considered in the serum, $V_{H}$ and ${\left[E\right]}_{H}$ are the volume of haemolysate and the concentration of the mineral considered in the haemolysate, $V_{F}$ is the final volume (sum of $V_{S}{+V}_{H}$), *CV* is the coefficient of variation for the determination of the mineral considered by ICP-MS and [Hb] is the concentration of haemoglobin in g L^−1^.

### 2.5. Data Analysis

All statistical analyses were carried out using SPSS for Windows (vs. 25, Armonk, NY, USA). The normality of data distribution was checked using the Kolmogorov–Smirnov test. The mineral element concentrations in whole blood and serum were compared using a *t*-test. The effect of graded haemolysis on mineral element concentration in serum was evaluated using a one-way ANOVA and, when significant, a post hoc Tukey’s multiple comparison test was applied to the data. The association between the estimated and the measured Fe concentrations in haemolysed serum samples was evaluated using the Pearson correlation coefficient after logarithmic transformation of the data. In all cases, a significance level of *p* < 0.05 was applied. Graphical outputs were obtained from Statgraphics Centurion XVI v. 18.1.12 (Statistical Graphics Corp., Rockville, MD, USA).

## 3. Results and Discussion

### 3.1. Mineral Concentrations in Paired Blood and Serum Samples

**Figure 1:**
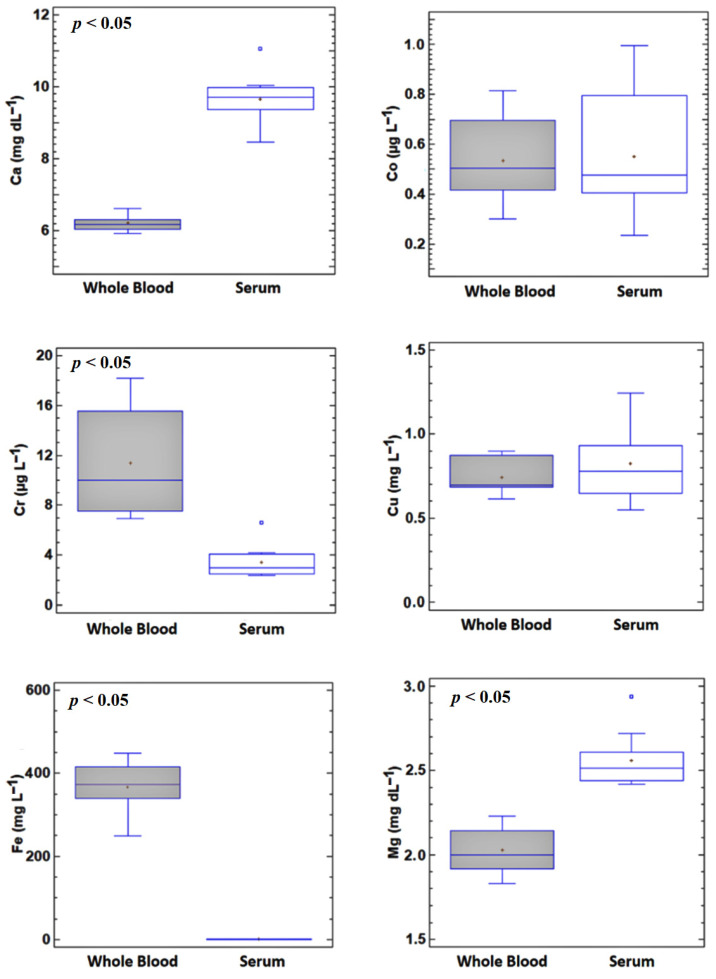

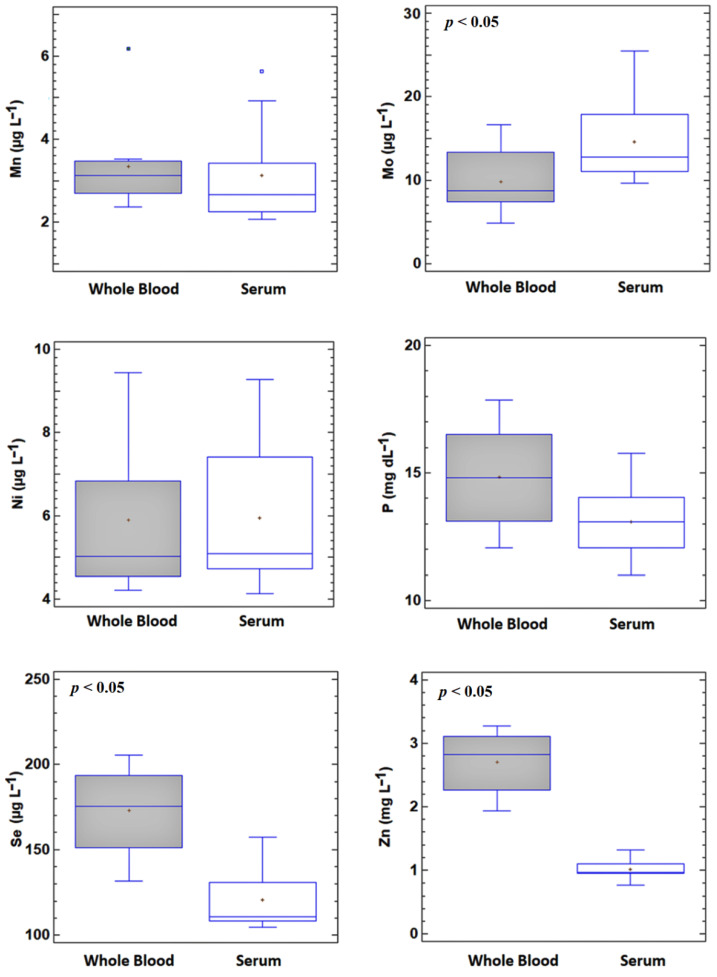
Box-and-whisker plots showing macro and microminerals in paired whole blood and serum samples (*n* = 10). *p* < 0.05 indicates a statistically significant difference between whole blood and serum values. Cr, Fe, Se and Zn are significantly higher in whole blood and Ca, Mg and Mo are significantly higher in serum.

The mineral concentrations in paired whole blood and serum samples (*n* = 10) are summarised in Figure 1. Iron was the most abundant element in whole blood (364 ± 64 mg L^−1^, ranging from 211 to 381 mg L^−1^), in concentrations approximately 300 times higher than those in serum. Whole blood Fe is mainly represented by Hb, which contains four Fe atoms per molecule [1]. The variations in the ratio of Fe in whole blood:serum can almost exclusively be attributed to the number and volume of erythrocytes. Physiological variations in red blood cells are observed among individuals, as well as in numerous pathological conditions, particularly those causing anaemia. Overall, the results obtained indicate that haemolysis can strongly influence the determination of the Fe status of cattle and could lead to the overestimation of Fe, although this error can potentially be corrected if the HD is calculated by considering that 1 g of haemoglobin contains 3.47 mg of Fe [29].

Chromium, Zn and Se concentrations were also significantly higher in whole blood than in serum, with whole blood:serum ratios of 3.73, 2.71 and 1.43, respectively. For all of these elements, the largest interindividual variations were found for the whole blood samples (Figure 1). Both Se and Zn are cofactors or essential intra erythrocyte enzymes that are closely involved in the immune system, which could explain the large individual variation within subjects. Selenium is associated with GPX1, the most abundant glutathione peroxidase in the body, and is also responsible for most of the blood Se concentration (erythrocyte Se usually represents 60–73% of the Se in whole blood in cattle [1,22]). Zinc is also essential for multiple intraerythrocytic enzymes such as superoxide dismutase, carbonic anhydrase and lactate dehydrogenase (LDH) [1,3]. The high concentration of LDH in red blood cells leads to its overestimation when measured in serum with HD values lower than 0.5 g Hb L^−1^ [23]. Regarding Cr, when present as Cr (III), it constitutes an essential mineral with beneficial influence on immune and antioxidative activity and energy metabolism [30,31] and is transported in serum due to its inability to penetrate red blood membranes [32]. By contrast, the hexavalent form Cr (VI) is highly toxic and carcinogenic [33] and has a high affinity for erythrocytes, where it is reduced to Cr (III) and bound to the β-chain of Hb [32]. In the present study, the higher Cr concentration in the whole blood samples than in the serum probably reflected a slight exposure of the subjects to environmental Cr (VI).

The whole blood concentrations of Mg, Mo and Ca were significantly lower than in serum (whole blood:serum ratios of 0.79, 0.65 and 0.66 respectively). The intraerythrocytic concentrations of these elements are low and, as they are mainly transported bound to plasma proteins in the blood, sample haemolysis would lead to underestimation of the concentrations in serum due to a dilution effect. The Mg and Mo concentrations showed similar inter-subject variability in whole blood and in serum, making it difficult to precisely predict its influence. However, the Ca concentrations in whole blood revealed very low inter-individual variation, suggesting that the effect of haemolysis could potentially be estimated by the analysis of haemolyzed serum. For the other elements, there were no significant differences between the concentrations in whole blood and serum, suggesting that haemolysis would have a negligible effect on the concentrations measured in serum.

The essential role of mineral elements in animal health and production, together with the high prevalence of mineral imbalances worldwide, has led to a large body of research on mineral element metabolism in the last few decades, including the establishment of precise reference values in serum and plasma (for reviews, see [1,16,17]). However, information of mineral element concentrations in whole blood is scarce, with the exception of Se. This is probably because Se deficiency is one of the most prevalent and costly trace element imbalances in livestock and the determination of Se in whole blood provides accurate, long-term information about the Se status of the animal [1,13]. Information about the effect of haemolysis on the determination of mineral status in cattle and other livestock species is also very limited. In a comprehensive review by Herdt and Hoff [17] on the use of blood to evaluate the mineral status in ruminants, apart from Fe, possible leakage from erythrocytes is only mentioned in relation to Zn and Mn. According to these authors, haemolysis or prolonged contact of the serum with the clot leads to the escape of Zn and Mn from red blood cells into the serum, thus producing a false increase in serum concentrations. However, these suggestions are based on studies in humans, in which the whole blood:serum ratios are different from those in bovine species. In humans, concentrations of both Mn [34] and Zn [24] in erythrocytes are 10–20 times higher than in plasma or serum, i.e., a ratio whole blood:serum in the range 5–10. In this study, whole blood Zn concentrations were around three times higher than in serum and no difference was observed for Mn. These results indicate that haemolysis may have less influence on the determination of Zn and particularly Mn in cattle than in humans. For all these reasons, it is important to known how and the degree to which haemolysis affects serum concentrations of minerals.

### 3.2. Estimation of the Haemolysis Threshold Affecting Mineral Concentrations in Serum

The haemolysis threshold (HTr) was calculated as described in Section 2.4 for each mineral element that showed a statistically significant difference in whole blood and serum. The HTr values obtained are presented in Table 3.

As expected, the mineral element with the lowest HTr was that of Fe, for which a minimal haemolysis degree, corresponding to a leakage of 0.015 g Hb L^−1^, significantly increased serum Fe concentrations. The HTr is below both the level at which it is considered that haemolysis can affect the values obtained in a blood test (0.5 g Hb L^−1^) and the lower Hb concentration where haemolysis is conventionally considered to begin, i.e., in the range 0.2–0.5 g L^−1^ [21,35,36]. Furthermore, this threshold of 0.015 g Hb L^−1^ is neither perceived by the human eye [37,38] nor by other methods proposed to assess haemolysis, such as the spectrophotometric method described by Killilea et al. [24], with a LOD of 1 g Hb L^−1^. However, considering that the Fe in erythrocytes mainly reflects the Hb content, accurate correction of serum samples with abnormally high Fe concentrations could be performed if the Hb concentration in serum was established.

Zinc was also affected by haemolysis, with an HTr of 1.74 g Hb L^−1^. In contrast to Fe, this level of haemolysis is visible and easily detected by the human eye [37,38]. In fact, samples with a haemolysis degree around this value are conventionally classified as “mildly haemolysed” [35]. In contrast to Fe, correction of this element should not be attempted. Erythrocyte Zn leakage cannot be precisely predicted, as the individual variability of Zn in whole blood and serum samples is wider and the difference between them in the physiological range is lower (Figure 1). As already stated, most of the Zn inside erythrocytes forms part of the enzymes playing a role in the immune and antioxidative system; consequently, it varies greatly depending on the individual.

The other elements yielded high HTr values, indicating that they are only influenced by high levels of haemolysis, specifically around 4 g Hb L^−1^ for Cr and 8 g Hb L^−1^ for Ca, Se and Mo. The red colour of serum samples with these high Hb concentrations can be easily detected by simple visual inspection. The upper limit of haemolysis commonly applied in haemolysis interference studies is 10 g Hb L^−1^ and the European Federation of Clinical Chemistry and Laboratory Medicine recommends rejecting samples with higher levels of haemolysis [39]. The haemolysis threshold for Mg greatly surpasses this acceptability criterion, with a value around 25 g Hb L^−1^ needed to cause a significantly decrease in the serum concentration. This finding contrasts with those of human studies, which report a statistically significant increase in Mg above 1.27–1.3 g Hb L^−1^ [19,23], measured using colorimetric methods. These apparently contradictory results reflect the different whole blood:serum ratios in humans (ca. 2; [40]) and cattle (0.79; as described in Section 3.1). Moreover, Mg concentrations are known to be higher in whole blood than in plasma/serum in other veterinary/livestock species such as sheep, goats and horses [41]; in these species, haemolysis could lead to the overestimation of Mg in haemolyzed samples, reinforcing the idea that the extrapolation of the results obtained in humans or other species can lead to wrong interpretations.

To our knowledge, there are no available data on the haemolysis thresholds that interfere with mineral element analyses in cattle and other livestock species. In human studies, the HTr has only been determined for Zn. As previously mentioned, the human whole blood:serum ratio for Zn is greater than in cattle; thus, the human threshold limit of 1 g Hb L^−1^ [24] is lower than the Zn-HTr established here. Regarding Fe determinations in serum, although the threshold has not been established, veterinary clinical pathologists are aware that a low haemolysis degree can bias results; thus, Herdt and Hoff [17] recommend that Fe should be measured in blood in the absence of haemolysis. However, this may be inconvenient, as Fe would have to be quantified in a specific test.

### 3.3. Effect of Haemolysis on Mineral Concentration in Serum

**Figure 2:**
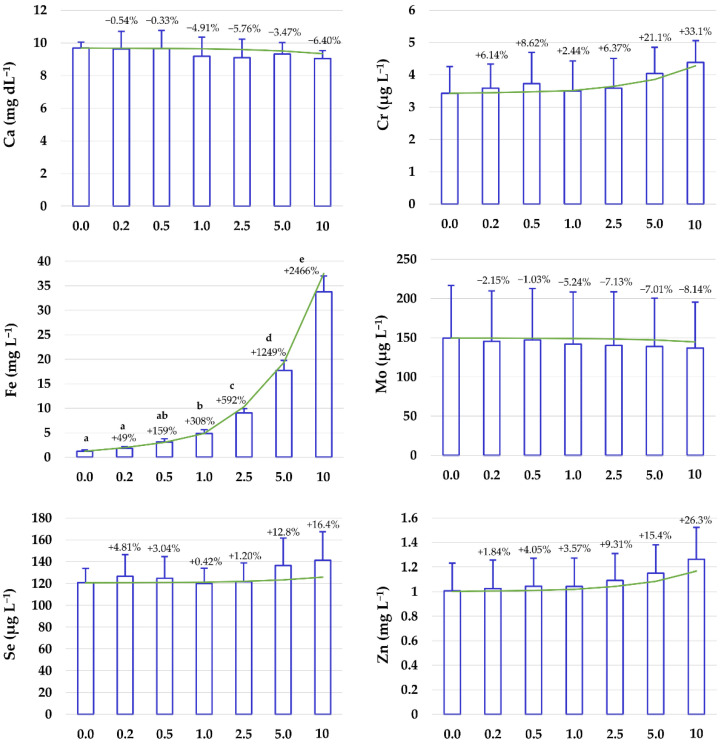
Effects of in vitro haemolysis on macro and micromineral concentrations in serum. Details of the haemolysate-spiked sample preparation are given in the text and summarised in Table 2. The green lines indicate the theoretical concentrations calculated considering the mean mineral concentrations in serum and whole blood. For each haemolysis degree, different letters indicate statistically significant differences. Percentage variation is expressed relative to the blank, non-spiked serum.

In the previous section, when establishing the HTr from which the haemolysis can potentially affect mineral concentrations in serum, only the influence of the technique variability (as the coefficient of variation) is considered. However, in practice, the interindividual variation in the mineral element concentrations in serum should also be considered. Therefore, with this aim, graded haemolytic serum samples (from 0.0% to 10% of haemolysis) were prepared, as explained in Section 2.1, and subjected to mineral analyses by ICP-MS. The results for all the minerals assayed, expressed as mean ± SD and percentage of variation compared to the blank-serum samples, are presented in Figure 2.

**Figure 3:**
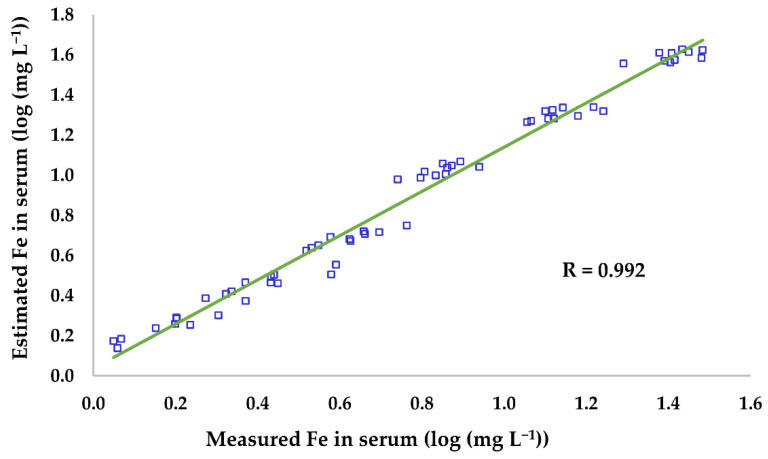
Correlation between the measured and estimated Fe concentrations (expressed as log (mg L^−1^)) in the graded haemolysed samples. Fe concentrations were estimated as the sum of the Fe concentrations in the blank, non-haemolysed samples and the Fe present in haemoglobin (1 g of haemoglobin contains 3.47 mg of Fe).

Iron was the only mineral that was significantly affected by the addition of haemolysate to the serum. Despite the good agreement between the theoretical percentage increase predicted by the model (Figure 2) and the measured serum Fe concentrations in the haemolysate-spiked samples for all the different degrees of haemolysis (from 0.2% to 10% haemolysate), statistically significant increases in Fe concentrations were only observed above 0.5% haemolysis (corresponding to a mean Hb concentration of 0.563 g L^−1^; range, 0.510–0.595 g L^−1^). These results were not surprising, as the variability of the analytical technique itself must be added to the inter-subject variability. Below this haemolysis degree, the Fe concentrations in most samples were within the physiological range (1.1–2.5 mg L^−1^) [17]. A haemolysis degree of 0.5 g Hb L^−1^ is similar to those reported in human medical studies to significantly modify Fe serum concentrations. Thus, e.g., Lippi et al. [19] observed significant differences from 0.6 g Hb L^−1^, using a colorimetric technique (ferrozine method) and Killilea et al. [24] established the significant HD at 0.5 g Hb L^−1^ with an ICP-OES method—a more sensitive technique. As already mentioned, the influence of HD on serum Fe concentrations can be accurately corrected by considering that 1 g of Hb contains 3.47 mg of Fe. The estimated concentrations of Fe in the haemolysate-spiked samples in this work (expressed as the sum of the Fe in the blank-serum samples plus the Fe contained in the Hb) were closely correlated with the measured Fe (R = 0.992; Figure 3).

However, no significant differences between the blank serum and the haemolysate-spiked samples were observed for any of the other minerals for which the concentrations were statistically significantly different in whole blood and serum (Figure 1) and whose theoretical haemolysis threshold was below the HD commonly accepted in clinical biochemistry (Table 2). Although a general trend (increase or decrease) was observed for all of these minerals, the data (including inter-subject and analytical variations) are within the physiological ranges of mineral concentrations in cattle (Ca, 9.7–12.4 mg dL^−1^; Mo, 2.0–35 µg L^−1^; Se, 0.065–0.14 mg L^−1^; Zn, 0.6–1.9 mg L^−1^) [17,28]. This prevents us from differentiating haemolyzed serum samples with mineral concentrations within the upper/lower part of the range (depending on whether the leakage leads to an increase/decrease in the element) with haemolysis from those falling within the opposite part of the range with no haemolysis. Therefore, preventing the haemolysis of samples is essential to produce reliable results when analysing cattle mineral profiles.

## 4. Conclusions

Significant differences were observed in the concentrations of most macro and microminerals, particularly Fe and Zn, measured in whole blood and serum. Such differences would lead to the overestimation of serum Fe concentrations in samples with a very low haemolysis degree (>0.015 g Hb L^−1^), as well as an overestimation of Zn in samples with a moderate haemolysis degree (>1.74 g Hb L^−1^). For the other minerals (Ca, Cr, Mo and Se) higher HD values would be needed to significantly modify the serum concentrations (>4 g Hb L^−1^). The broad inter-subject variability within the physiological ranges for most elements hinders the differentiation of valid and haemolysis-biased results; therefore, preventing the haemolysis of samples is indeed essential to yield reliable results. The recommended haemolysis acceptability thresholds are 2 g Hb L^−1^ for Zn, 4 g Hb L^−1^ for Cr and 8 g Hb L^−1^ for Ca, Se and Mo. As Fe alteration occurs at very low HD (0.015 g Hb L^−1^), Hb should be quantified to correct Fe results based on the HD.

## Figures and Tables

**Table 1:** Sample preparation for determination of the haemolysis degree in vitro.

Sample Preparation (per 1 mL)	Haemolysis Degree (HD) (%)						
	0.0	0.2	0.5	1.0	2.5	5.0	10
Serum (mL)	1.000	0.998	0.995	0.990	0.975	0.950	0.900
Haemolysate (mL)	0.000	0.002	0.005	0.010	0.025	0.050	0.100
HD (g Hb L^−1^)							
mean	0	0.225	0.563	1.13	2.81	5.63	11.3
range		(0.204–0.238)	(0.510–0.595)	(1.02–1.19)	(2.55–2.98)	(5.10–5.95)	(10.2–11.9)

**Table 2:** Results of the analytical quality control program.

Element	Detection Limit (µg L^−1^)	Animal Serum NIST 1598a		Spiked Samples
		Certified Value (µg L^−1^) ^1^	Recovery (%)	Recovery (%)
Ca	0.020	96 ± 7	95	99.8 ± 5.2
Co	0.008	1.24 ± 0.07	100	91.1 ± 1.7
Cr	0.093	0.33 ± 0.08	ND ^3^	96.4 ± 12.0
Cu	0.130	1580 ± 90	105	96.1 ± 5.7
Fe	0.147	1680 ± 60	114	95.8 ± 13.1
Mg	0.050	−		96.1 ± 5.7
Mn	0.014	1.78 ± 0.33	108	88.8 ± 4.4
Mo	0.089	5.5 ± 1.0	96	86.1 ± 8.7
Ni	0.112	0.94 ± 0.18	107	106 ± 5.0
P	0.017	(140) ^2^	99	101 ± 3.0
Se	0.023	134.4 ± 5.8	118	98.1 ± 5.2
Zn	0.005	880 ± 24	108	95.6 ± 6.7

^1^ Except for Ca, Mg and P, which are expressed in mg L^−1^; ^2^ in brackets, there are only indicative values; ^3^ ND, not detected.

**Table 3:** Estimation of the haemolysis threshold (HTr) affecting serum mineral element concentrations (only for those elements showing statistically significant differences between whole blood and serum measurements).

Element	[*E*]_Serum_ (mg L^−1^)	[*E*]_Haemolysate_ (mg L^−1^)	*CV*_technique_ (%)	HTr (g Hb L^−1^)
Ca	96.8	62.2	2.59	8.12
Cr	0.003	0.012	8.96	4.07
Fe	1.20	364	4.07	0.015
Mg	25.7	20.2	4.72	24.7
Mo	0.015	0.010	2.48	8.41
Se	0.120	0.173	3.28	8.48
Zn	1.003	2.664	2.57	1.74

## Data Availability

No new data were created or analyzed in this study. Data sharing is not applicable to this article.
